# Kimel Family Centre for Brain Health and Wellness: Protocol for a Naturalistic Longitudinal Personalized Multidomain Preference Dementia Risk Reduction Trial

**DOI:** 10.1002/alz.085754

**Published:** 2025-01-09

**Authors:** Nicole D. Anderson, Danielle D'Amico, Shlomit Rotenberg, Jenna Gillen, Daniel Moore, Brian Tan, Malcolm Binns, Melanie Santarossa, Howard Chertkow, The Canadian Consortium on Neurodegeneration in Aging (CCNA), CAN‐THUMBS UP

**Affiliations:** ^1^ University of Toronto, Toronto, ON Canada; ^2^ Baycrest Academy for Research and Education, Toronto, ON Canada; ^3^ Baycrest Academy of Research and Education, Toronto, ON Canada

## Abstract

**Background:**

The Kimel Family Centre for Brain Health and Wellness is a research‐driven community centre testing the efficacy of a naturalistic longitudinal personalized multidomain preference dementia risk reduction intervention on dementia risk and cognition. The objective of this protocol is to validate this approach by following people for two years.

**Method:**

Participants (*n* = 325) will be 50 years of age or older, without a diagnosis of dementia, and sufficiently fluent in English to complete the assessments and understand program instructors. Participants will receive a comprehensive dementia risk assessment, including both nonmodifiable and modifiable risk factors, from which they will receive a Personalized Dementia Risk Report and Program Strategy, indicating health conditions increasing dementia risk, and their risk level in five risk domains: physical activity, brain‐healthy eating, cognitive engagement, social connections, and mental wellbeing. Participants will select programs to meet their Personalized Program Strategy. We will examine the effects of this program on cognition (MoCA and Cogniciti’s Brain Health Assessment) and risk in the five domains, as a function of adherence (attendance), compared to those of 300 individuals who will complete the Canadian Consortium on Neurodegeneration’s CAN‐THUMBS Up online, educational program on modifiable dementia risk factors, called Brain Health PRO.

**Result:**

We expect better maintenance of cognition and reduction in the five dementia risk domains in Kimel Family Center participants, compared to Brain Health PRO participants.

**Conclusion:**

There is an urgent need to develop scalable multidomain programs for dementia prevention. This innovative approach overcomes a number of limitations present in prior multidomain dementia prevention trials. Once validated, the approach will be scaled to other community centres, to benefit a greater number of geographically dispersed individuals.

